# Some Observations on Hemoptysis

**Published:** 1905-01

**Authors:** Guy H. Fitzgerald

**Affiliations:** Albuquerque, N. M.


### SOME OBSERVATIONS ON HEMOPTYSIS.

BY GUY H. FITZGERALD, A.M., M.D., ALBUQUERQUE, N. M.

Control of blood-pressure is the one great essential in the treatment of hemoptysis. Blood-pressure varies within certain physiologic limits and many seemingly trivial means may be quite useful in keeping it low, or in avoiding sudden increases. For instance any physical exertion, the presence of much food in the stomach, a sudden high fever, severe pain, anxiety, fear or mental excitement, all contribute to a pressure higher than normal. The vasomotor mechanism is so responsive to a great variety of impressions that blood-pressure readings by mechanical means are at best but approximate; however, careful repeated observations will give fairly accurate records. Records made by a Riva-Rocci sphygmomanometer form the basis of the observations cited in this paper.

That altitude is not a causative factor of any moment in producing pulmonary hemorrhage is evident from observations made on both sick and healthy people on arrival at an elevation of 5,000 feet. The readings show the blood-pressure lower than that of old residents of the same age and sex, and lower than the point to which it rose after some weeks' residence. Many cases of hemoptysis occur at this altitude, but the proportion of hemorrhages to the number of cases is no greater here than elsewhere. On going from a low to a high altitude the atmospheric pressure on the general body surface is lessened and general blood-pressure falls in proportion. Nearly all persons on reaching a high altitude experience some slight disturbance. The pulse-rate is usually increased and blood-pressure lowered. The dizziness, shortness of breath, headache and nausea often experienced, are partly due to disturbance of the factors which maintain blood-pressure. In the great majority of cases the heart and vasomotor system promptly meet the demand for more work and compensation is soon established, after which blood-pressure again reaches the normal level.

Observations on blood-pressure made before, during and after hemorrhage usually showed a decline from the normal pressure during and after the bleeding. Only in nervous, excited or fearful subjects did the pressure remain high, or rise to a higher point than was observed before the hemorrhage. Four patients who registered a practically normal pressure for some weeks preceding a hemorrhage, during and immediately after the bleeding, showed a decided fall in pressure, though the loss of blood in no instance was great. None of these patients were of a nervous temperament. Two nervous patients, who previously registered normal pressures, 145 and 155 mm., on having hemorrhages showed erratic variations in pressure, which ranged from 125 to 190 mm. For a week following there were occasional hemorrhages which occurred when the pressures were highest. Both cases were extremely excitable, and fearful of death. These variations in pressure were most probably of psychic origin, since other factors could be largely excluded.

The psychic effect of hemoptysis on blood-pressure varies with the patient's temperament. The more usual course is for blood-pressure to fall owing to the depression which such an accident causes, even in the phlegmatic. Nature thus lowers blood-pressure and favors thrombosis. This decline in pressure is noted even when the loss of blood is small. A hard, rapid, high-tension pulse with high blood-pressure is found in the nervous, excited or hysterical subjects, and in these cases the control of blood-pressure is often difficult. Reassurance by the physician, and confidence inspired by his manner and work, may be of the greatest value in allaying excitement and fear, and thus materially aid in lowering blood-pressure. Nerve sedatives, as bromides, in large doses, are often more effective in lowering blood-pressure in these subjects than the direct circulatory depressants. When the blood-pressure continues high during a hemorrhage, the cause is usually psychic and suggestion and nerve sedatives will give the best results. Often in cases where the nervous element is least suspected, it remains a concealed cause of high pressure. A patient may give every assurance that he is not worried or anxious, and may seemingly be fully reassured by the advice and counsel of his physician, and yet underneath it all his secret anxiety may do more harm than that of the patient who is open and frank about it. Too much stress can not be laid on the patient's mental make-up, and in gaining his fullest confidence. No accident causes greater anxiety and fear than hemoptysis even in the care-free and phlegmatic. It is regarded as a most dangerous sign and causes more mental distress than any other symptom in phthisis. Hence the wisdom of preparing patients beforehand for this complication by careful explanation of what a hemorrhage means and that it is not usually serious. A patient so prepared is able to face a hemorrhage with a calmness and quiet which aids greatly in lessening its severity.

Theoretically the nitrites—such as amylnitrite, or nitro-glycerin —by lowering general blood-pressure should indirectly lower the pulmonic pressure and thus favor clotting. According to Sollman, vasomotor drugs have little or no direct effect on the pulmonic blood-pressure. In practice these drugs seem to give little result in the control of pulmonary hemorrhages, even when the effect is pushed. This is particularly true of nervous subjects in whom the blood-pressure varies greatly. If the loss of blood has been great, full doses of the nitrites may produce an alarming collapse which it is hard to overcome.

The treatment of hemoptysis will vary with the type of bleeding. If an area of congestion or hyperemia is causing slight bleeding, if the sputum is but tinged with blood, or if there are but a few mouthfuls of bright frothy blood, rest in bed may be all that is required in addition to careful directions about exertion for some weeks to come. Bleeding of this type usually subsides within a short time. If the hemorrhage is more severe, or if a vessel has ruptured, a hypodermic of morphia and atrophia is best given at once. The danger of having blood pass into other portions of the lungs, owing to lessened sensitiveness of the bronchial tubes, is not very great. These patients often take large doses of morphia without subsequent ill effects. The one great essential is to do everything possible to favor thrombosis. Absolute physical rest and mental quiet are imperative if a low blood-pressure is to be favored. Morphia aids greatly in producing physical rest and no one drug is so effective in allaying mental excitement. The patient is best placed in a semirecumbent position, in which he can expectorate with the least effort; he should not speak above a whisper, and should make no movements which are not absolutely necessary. As physical effort raises blood-pressure, cough should be controlled at all hazards. Codeia or morphia are most effective. The sudden mechanical distention of the lung in the act of coughing may readily dislodge a newly-formed clot; or the additional increase in blood-pressure, caused by the physical effort of coughing, may force out a thrombus and renew the bleeding. A hard coughing fit may cause a rise from 90 to 100 mm. in blood-pressure. No physical examination, especially percussion or deep breathing, should be allowed, as nothing of benefit can be gained thereby. Since solid food or large amounts of liquid food in the stomach will cause a rise in blood-pressure, nourishment is best given in small amounts and in liquid form so long as there is any sign of bleeding. If high, blood-pressure should be lowered, and if the bleeding is not very severe, a saline laxative may aid greatly; if the bleeding is severe, it should be withheld, since the efforts in bowel movement may raise blood-pressure. Morphia will indirectly help in lowering blood-pressure by its mental as well as physical action. The patient's fears should be allayed if possible. Ice-bags over the heart, if its action is tumultuous, may do good. Common salt and small pieces of cracked ice by mouth are beneficial in the way of suggestion at least. When acute pleurisy complicates hemorrhage the bleeding is often intractible; sudden sharp pain almost invariably raises blood-pressure, and the pleurisy may be painful enough to keep the pressure high. In addition to morphia, strapping may be necessary if the pleurisy and bleeding points are not on opposite sides. A high fever, or even a sudden rise of a degree or two in temperature, usually causes a rise in blood-pressure, and hence if fever is present it should be controlled. Antipyretics may be needed to lower the fever and indirectly the blood-pressure. Bromides and nerve sedatives are of great value in the nervous. If blood-pressure continues high, in spite of these measures, some of the circulatory depressants, as veratrum or aconite, may be used. However, they are not so efficient in lowering blood-pressure in the excited or hysterical subject unless pushed to almost the toxic stage. Amylnitrite or nitroglycerin give better results with less danger. Overdrugging is common in the treatment of hemoptysis, and especially is this true in the use of circulatory depressants, when blood-pressure is already sufficiently low. They are best held in reserve until other and less severe measures fail.

Ergot has no value in pulmonary hemorrhages, either theoretically or practically. Observations made on patients taking large doses invariably showed a rise in blood-pressure with no lessening of the bleeding. Heroic doses, taken on their own volition for twenty-four and thirty-six hours by two patients, resulted in a blood-pressure of 210 mm. in one case and 185 mm. in the other, with a constant increase in the bleeding. The one subject was of a nervous temperament, which may have contributed to the high pressure; but the other was phlegmatic and not at all excitable. In no instance in a large series of cases could the control of hemorrhage be attributed to ergot. Adrenalin by mouth taken in small repeated doses, or in large single doses, failed to control bleeding. Its effect on blood-pressure in these cases was not marked, though as a rule there was a slow gradual rise in pressure. When given in heroic doses in desperate cases, the results were not satisfactory. Too much dependence is often placed on ergot or adrenalin as specifics in directly controlling hemorrhages. Neither drug has much effect on pulmonary hemorrhage, and certainly the rise in general blood-pressure following their use will tend to increase the bleeding.

Gelatin or calcium-chlorid given in cases which were subject to frequent small oozings, rather than to large hemorrhages, failed to materially check the bleeding. In severe cases, where immediate results were demanded, their action was too slow. In cases where the drugs could be given for some time, rather as a prophylatic than for the immediate control of bleeding, no great benefit could be noted. Neither drug is objectionable, since they have no action on blood-pressure. In the same category fall gallic and tannic acids, hydrastis, alum, dilute sulphuric acid, acid, and other drugs given with the idea of increasing the coagulability of the blood. Digitalis is dangerous because it causes a decided rise in blood-pressure.

When the hemorrhage has been profuse, and the patient is exsanguinated, normal saline solution by hypodermoclysis may be indicated. It must be given with caution since blood-pressure may be raised to a dangerous level. Small amounts, repeated as required, are safer than one large injection. That the pulse is soft and the blood-pressure is low is beneficial rather than otherwise, since the greatest danger lies in a too high rather than a too low blood-pressure.

The best treatment of hemoptysis requires: (1) absolute physical rest; (2) mental quiet and relief from fear and anxiety; (3) morphin and atropia in sufficient dosage to insure both the preceding; (4) control of cough, fever and pleuritic pain and careful attention to diet; (5) suggestive measures as ice-caps over the heart, salt and cracked ice by mouth, etc.; (6) free use of bromides and nerve sedatives in the nervous; (7) nitrites or veratrum when high blood-pressure persists; (8) care in not overdrugging or in placing reliance on specifics as ergot or adrenalin; (9) hypodermoclysis with normal saline when indicated.—*The Cleveland Medical Journal.*

